# Morphometric measurements of dragonfly wings: the accuracy of pinned, scanned and detached measurement methods

**DOI:** 10.3897/zookeys.276.4207

**Published:** 2013-03-08

**Authors:** Laura Johnson, Beth L. Mantle, Janet L. Gardner, Patricia R. Y. Backwell

**Affiliations:** 1 Research School of Biology, The Australian National University, 116 Daley Road, Canberra, ACT 0200, Australia; 2 Australian National Insect Collection, CSIRO Ecosystem Sciences, GPO Box 1700, Canberra, ACT 2601, Australia; 3 current address: School of Biological Sciences, Monash University, Melbourne, Victoria 3168

**Keywords:** Digitization, entomological collections, morphometrics, museum collections, dragonflies

## Abstract

Large-scale digitization of museum specimens, particularly of insect collections, is becoming commonplace. Imaging increases the accessibility of collections and decreases the need to handle individual, often fragile, specimens. Another potential advantage of digitization is to make it easier to conduct morphometric analyses, but the accuracy of such methods needs to be tested. Here we compare morphometric measurements of scanned images of dragonfly wings to those obtained using other, more traditional, methods. We assume that the destructive method of removing and slide-mounting wings provides the most accurate method of measurement because it eliminates error due to wing curvature. We show that, for dragonfly wings, hand measurements of pinned specimens and digital measurements of scanned images are equally accurate relative to slide-mounted hand measurements. Since destructive slide-mounting is unsuitable for museum collections, and there is a risk of damage when hand measuring fragile pinned specimens, we suggest that the use of scanned images may also be an appropriate method to collect morphometric data from other collected insect species.

## Introduction

Digitized imaging of museum collections is becoming increasingly commonplace. Large-scale imaging of biological collections, particularly those of insects and plants, are currently being undertaken at many museums (Beaman and Cellinese 2012). Digitized collections have many advantages: they make collections globally accessible; they decrease the risk of damage associated with accessing and handling specimens; and they last indefinitely. Many museums image individual specimens (Tegelberg et al. 2012; Flemons and Berents 2012) while others use whole-drawer imaging, especially of insect collections (Mantle et al. 2012; Bertione et al. 2012; Dietrich et al. 2012; Schmidt et al. 2012). The advantage of whole-drawer imaging over imaging and/or databasing individual specimens is the speed with which it can be done, thereby allowing rapid, large-scale digitization of whole collections. There is also no need to handle specimens that are sometimes very fragile and/or valuable.

If the method of digitization is appropriate, it might also be possible to use the images for the collection of morphometric data. The North Carolina State University GigaPan project images whole-drawers of insect collections but produces images with curvature and distortion around the edges, precluding their use in morphometric analyses (Bertone et al. 2012). There are two systems, however, that have overcome the distortion problem by using a camera that moves over the entire drawer, takes multiple scans and then stitches them together. The DScan is used by the Munich Zoologische Staatssammlung in Germany (Schmidt et al. 2012) and the SatScan system is used by both the Australian National Insect Collection (ANIC) and Natural History Museum, London (Mantle et al. 2012; Blagoderov et al. 2012). Both DScan and SatScan have the potential to create images that can be used for morphometric studies of insect specimens, but the accuracy of these systems has not been investigated to date. Validation is desirable, because there are obvious advantages to measuring scanned images rather than pinned specimens. Preserved insects can be extremely fragile and are easily damaged during handling, which is problematic because most museum specimens are very valuable and often irreplaceable. Consequently, hand measurements of pinned specimens are often restricted to highly experienced museum staff, thereby limiting the scope of studies undertaken.

Here we examine whether accurate morphometric measurements can be obtained from digital images collected during whole-drawer scans using the SatScan system. We selected dragonfly wings because they are particularly difficult insects to handle and pin. Furthermore, the wings may be set at an angle to the horizontal, or with slight curvature over the length of the wing; this preparation artifact could limit the usefulness of scanned images for morphometric analysis since the 2D images might systematically underestimate wing length (Mantle et al. 2012). We suggest that the most accurate method for measuring dragonfly wings is to remove them from specimens and mount them between microscope slides, thereby eliminating measurement error due to curvature. In this study, we used a collection of dragonflies, each of which we measured in four different ways. (1) We used calipers to hand measure the wing length of pinned specimens removed from their drawers; (2) we scanned specimen drawers and used the digitized images to measure the wings, and (3, 4) we removed wings from specimens and slide-mounted them in preparation for hand measuring (first with their identifier labels visible and then with their labels obscured). We then compared the measurements from slide-mounted specimens to those taken from pinned and scanned wings respectively.

## Methods

We measured the right forewings of 71 assorted, unidentified specimens of dragonflies. Each wing was measured a total of twelve times with three repeated measures for each of four different methods: pinned specimens, scanned images, and two sets of measures on slide-mounted wings. The wing was measured from the first cross-vein (ax0; Bechly 1996) to the furthest point of the wing tip. For consistency, all measurements were made by a single researcher (LJ).

### Pinned measurements

We successively removed sets of three pinned specimens from their collection drawer and pinned them on separate foam blocks. We measured the right forewing in situ, using the tips of the lower jaws of digital calipers, recording to an accuracy of 0.01mm. We took three measurements for each specimen by sequentially measuring each individual in the set of three so that no single individual was measured twice in a row. The calipers were closed and re-zeroed after each measurement.

### Scanning

We scanned whole drawers of specimens using a SatScan^TM^ imaging system developed by SmartDrive Ltd. The system uses a Basler A631FC ½” CCD camera with a 0.16x telecentric lens that moves along rails positioned above the drawer (Mantle et al. 2012). This minimizes the distortion and provides images with no parallax artifacts. The camera captures 200-400 ‘tile’ images at precise positions, and these are then ‘stitched’ together to produce a single high-resolution image of the entire drawer (see Mantle et al. 2012). External light is excluded with shields and a controlled light source is provided by internal fluorescent tubes (Mantle et al. 2012). Prior to scanning, the dragonflies were not repositioned within their drawer so that their wings were horizontal. The final image was analyzed using SigmaScan^TM^ software. Three measurements were made of each wing using a digital pointer to mark the end points of the measurement, recording to an accuracy of 0.01mm.

### Slide measurements

After hand measuring then scanning the pinned specimens, we used fine forceps to remove the right forewing of each specimen under a 10x magnification microscope lens. The detached wing was placed between labeled glass slides with a drop of water for cushioning. We measured the flattened wings in the same way as the pinned specimens, using the same calipers, recording to an accuracy of 0.01mm.

### Slide-blind measurements

We re-measured the slide-mounted wings but with the identifier label replaced by a random specimen number to ensure that no subconscious bias could affect the measurements. The wings were otherwise measured as described above.

### Statistics

To compare the estimated means between the four measurement types we ran a mixed model with measurement type as a fixed factor and specimen identity as a random factor (to control for repeated measurements). We estimated repeatability for each measurement type using a one-way ANOVA and calculated the intra-class correlation (r_I_) following the methods of Lessells and Boag (1987). All statistical tests were conducted using SPSS 19.0.

## Results

**Figure 1.**
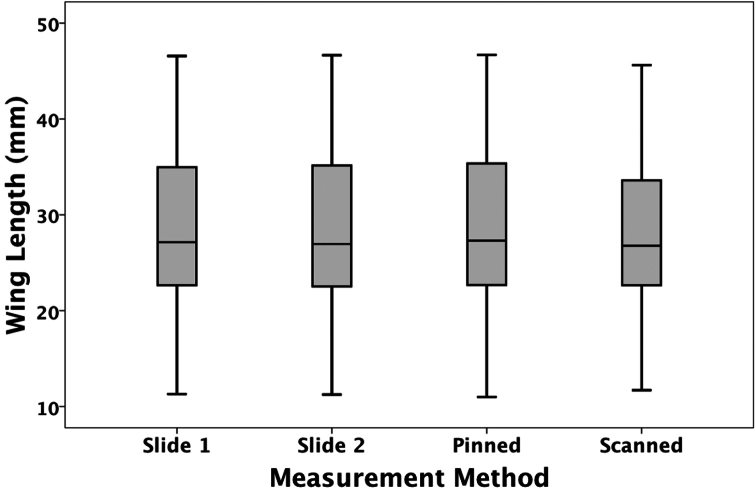
Estimated mean forewing lengths obtained using the four different measurement methods. Slide 1 = identifier visible; slide 2 = identifier obscured. The median, quartiles and range are shown.

The estimated mean forewing lengths obtained using the four different methods were (Mean ± SE (in mm): pinned: 29.38 ± 1.04; scanned: 28.77 ± 1.04; slide-mounted: 29.24 ±1.04; blind slide-mounted: 29.24 ±1.04; all n = 71) (Figure 1). There was a significant difference in estimated mean size among the four measurement types (F_3,778_ = 58.16; P<0.001). All pair-wise differences were significant (Bonferonni tests, all P < 0.005) except for that between the two slide-mounted measures (i.e. regardless of whether the label was visible or hidden) (P = 0.88).

For all four measurement types, the three repeated measures for each specimen were highly repeatable (Lessells and Boag 1987) (pinned: F_2,68_ = 15163.79, P < 0.001, r_I_=0.999; scanned: F_2,68_ = 9630.54, P < 0.001, r_I_=0.999; slide-mounted: F_2,68_ = 7389.77, P < 0.001, r_I_=0.999; blind slide-mounted: F_2,68_ = 17882.37, P < 0.001, r_I_=0.999 ) (Figure 1).

Based on the assumption that slide-mounting gave the most accurate measure of wing length, we tested whether there was a significant difference in the extent to which measurements from pinned and scanned specimens, respectively, deviated from those obtained from slide-mounted specimens. To do this, we re-ran two separate mixed models, first comparing the treatments of slide-mounted and pinned, then comparing slide-mounted and scanned. For each model we then calculated the absolute value of the effect size (i.e. the standardized magnitude of the difference between the slide mounted and alternate treatment). The effect size *r* was calculated from the *F* statistic using a standard formula (Koricheva et al. 2013). We then compared the effect sizes using a standard test to compare two correlation coefficients (Zar 1984). There was no significant difference between the two effect sizes (Z = 0.543, P = 0.587). The pinned and scanned measurements showed equivalent degrees of variation compared to those from slide-mounted measurements.

## Discussion

Slide-mounted wings were measured with their identifying labels visible (three measurements) and with their labels hidden (three measurements). These two sets of readings were statistically identical, and the measurements were highly repeatable. This suggests that the measurements on slide-mounted wings were extremely precise, and may be regarded as the most accurate method to measure size. By removing the wing and mounting it between microscope slides, the wing is flattened and the potential problems of curvature and wing angle are eliminated. This makes it easier to obtain an accurate measurement of maximum length. Unfortunately this is a very destructive method, and is unsuitable for most museum collections.

The two alternative methods for measuring dragonfly wings, hand measuring pinned specimens using calipers and digital measurement of (whole-drawer) scanned images, were also highly repeatable. In both cases, however, the estimated means for wing length differed from that for slide-mounted wings. The pinned specimens yielded the largest and the scanned images the smallest estimated mean. It is not surprising that scanned images resulted in smaller readings since the two dimensional image does not allow compensation for wing curvature or the angle at which the wings are set, relative to the insect’s body. In addition, the angle at which specimens are positioned within drawers will lead to foreshortening if they are not set parallel to the camera lens. It is less clear, however, why pinned specimens produce larger measurements. It is difficult to measure dragonfly wings *in situ*: they are fragile and the calipers need to be moved very carefully to avoid touching the specimen. There might be a bias when measuring curved or angled wings to compensate for this problem, which results in a slight overestimation of wing length.

Given that the measurements from pinned specimens and those from scanned images were equally inaccurate compared to those of slide-mounted wings; we suggest that there is no advantage in measuring pinned specimens over scanned images. There is a cost, however, to measuring pinned specimens: the procedure is very time-consuming and has greater risk of damaging the specimens compared to the use of scanned images. We therefore suggest that scanned images are an appropriate way of measuring specimens, particularly those that are fragile like dragonflies.

It is important to note that we selected dragonfly wings for this study because they are fragile and most likely to show preparation artifacts that decrease measurement accuracy (e.g. wing curvature). Measuring sturdier structures like the elytra of beetles is likely to be more accurate, using both digital measures of scanned images and hand measuring pinned specimens. Pinned specimens might provide the more accurate estimate in such cases, because calipers can come into contact with the structure with less fear of damaging the specimen. This might reduce the risk of over measurement that we suspect affected our pinned specimen wing data. In addition, the inherent and random error arising from the angle at which specimens are secured within drawers is eliminated.

Despite these potential advantages, however, repeated handling of specimens will inevitably lead to damage via breakages or the removal or abrasion of fine structures (e.g. antennae). Accordingly, the relative risks of the handling specimens must be considered in the context of measurement error when choosing appropriate study methods. Our study shows that the use of scanned images is a better method in the case of fragile specimens and should be routinely considered as a technique in any museum study.

## Conclusion

Measuring detached, slide-mounted dragonfly wings is the most accurate method for morphometric studies but is obviously unusable for museum specimens. Here we show that, for dragonfly wings, hand measurements of pinned specimens and digital measurements of scanned images are equally accurate relative to slide-mounted measurements. Hand-measuring pinned specimens carry a risk of damaging the insects. We therefore suggest that the use of whole-drawer scanned images is an appropriate method to collect morphometric data on dragonfly wings. For other collected insects, we suggest this method should be considered as an alternative to hand measuring pinned specimens when the measurement precision required and the fragility of the specimens are taken into account.

## Appendix

Morphometric measurements of dragonfly wings (doi: 10.3897/zookeys.276.4207.app) File format: Microsoft Excell document (xls).
